# Supplementary material for: Personalised immunotherapy in sepsis: a scoping review protocol
Source: BMJ Open. 2022 May 9;12(5):e060411. doi: 10.1136/bmjopen-2021-060411 (PMC9086601; doi:10.1136/bmjopen-2021-060411)
Supplement: Supplementary data [file bmjopen-2021-060411supp001.pdf]

Supplementary online content

Personalized immunotherapy in sepsis: a scoping review protocol

M.A. Slim*, N. van Mourik*, J.C. Dionne, S.J.W. Oczkowski, M.G. Netea, P. Pickkers, E. J. Giamarellos-Bourboulis, M.C.A. Müller, T. van der Poll, W.J. Wiersinga, A.P.J. Vlaar, L.A. van Vught
* Both authors contributed equally.

**Content**	**Page**
Supplementary Methods	2
Supplementary Table 1.	4
Supplementary Table 2.	5
Supplementary Table 3.	6
Supplementary Table 4.	7
Supplementary Table 5.	8
Supplementary Table 6.	9

Slim MA, *et al. BMJ Open* 2022; 12:e060411. doi: 10.1136/bmjopen-2021-060411

Supplementary Methods. The full search string

PubMed
2021 May 04

2444 hits:
("Sepsis"[Majr] OR sepsis[tiab] OR septic[tiab])

AND

("Immunotherapy"[Mesh] OR "Precision Medicine"[Mesh] OR "Immunosuppression"[Mesh] OR "Anti-Inflammatory Agents/therapeutic use"[Mesh] OR "Anti-Infective Agents/therapeutic use"[Mesh] OR "Glucocorticoids"[Mesh] OR "Hydroxycorticosteroids"[Mesh] OR "Anticoagulants/therapeutic use"[Mesh] OR "Cytokines/antagonists and inhibitors"[Mesh] OR "Cytokines/therapeutic use"[Mesh] OR "Granulocyte Colony-Stimulating Factor/therapeutic use"[Mesh] OR "Granulocyte-Macrophage Colony-Stimulating Factor/therapeutic use"[Mesh] OR "Immunoglobulins, Intravenous/therapeutic use"[Mesh] OR "Programmed Cell Death 1 Receptor/therapeutic use"[Mesh] OR "Toll-Like Receptors/therapeutic use"[Mesh] OR "Polymyxin B/therapeutic use"[Mesh] OR "Thymosin/therapeutic use"[Mesh] OR "Protein C/therapeutic use"[Mesh] OR "Mesenchymal Stem Cells"[Mesh] OR immunotherap*[tiab] OR interleukin*[tiab] OR interferon[tiab] OR toll-like-receptor agonist*[tiab] OR anti-endotoxin compound*[tiab] OR blocking complement*[tiab] OR activated vitamin C[tiab] OR mesenchymal stem cell*[tiab] OR immune checkpoint inhibitor*[tiab] OR extracorporeal blood removal[tiab] OR corticosteroid*[tiab])

AND

("Clinical Trial" [Publication Type] OR clinical trial*[tiab] OR randomized trial*[tiab] OR randomized trial*[tiab] OR controlled trial*[tiab])

NOT

(("Child"[Mesh] OR "Infant"[Mesh] OR "Pediatrics"[Mesh] OR child*[tiab] OR infan*[tiab] OR newborn*[tiab] OR neonat*[tiab] OR baby[tiab] OR babies[tiab] OR pediatr*[tiab] OR paediatr*[tiab]) NOT "Adult"[Mesh])

NOT

("Editorial"[Publication Type] OR "Letter"[Publication Type] OR "News"[Publication Type] OR "Comment"[Publication Type] OR "Review" [Publication Type] OR "Case Reports"[Publication Type] OR "letter*"[Title] OR "comment*"[Title] OR editorial[ti])

NOT

(("Animals"[MeSH Terms] OR "Animal Experimentation"[MeSH Terms] OR "animals, laboratory"[MeSH Terms] OR "models, animal"[MeSH Terms] OR "animal*"[Title/Abstract] OR "rat"[Title/Abstract] OR

"rats"[Title/Abstract] OR "mice"[Title/Abstract] OR "mouse"[Title/Abstract] OR "dog"[Title/Abstract] OR "dogs"[Title/Abstract] OR "pig"[Title/Abstract] OR "pigs"[Title/Abstract] OR "cow"[Title/Abstract] OR "cows"[Title/Abstract] OR "monkey"[Title/Abstract] OR "monkeys"[Title/Abstract] OR "horse"[Title/Abstract] OR "horses"[Title/Abstract] OR "sheep"[Title/Abstract] OR "ovine"[Title/Abstract] OR "lamb"[Title/Abstract] OR "lambs"[Title/Abstract] OR "goat*"[Title/Abstract] OR "swine"[Title/Abstract] OR "porcine"[Title/Abstract] OR "pup"[Title/Abstract] OR "pups"[Title/Abstract] OR "canine"[Title/Abstract] OR "bitch*"[Title/Abstract] OR "beagle"[Title/Abstract] OR "feline"[Title/Abstract] OR "rodent*"[Title/Abstract] OR "rabbit*"[Title/Abstract] OR "murine"[Title/Abstract] OR "ape"[Title/Abstract] OR "apes"[Title/Abstract] OR "gorilla"[Title/Abstract] OR "gorillas"[Title/Abstract] OR "catfish"[Title/Abstract]) NOT "Humans"[MeSH Terms])

Cochrane Central Register of Controlled Trials
Issue 4 of 12, April 2021

ID Search Hits

#1 (sepsis or septic):ti,ab,kw 13698

#2 (immunotherap* OR interleukin* OR interferon OR toll-like-receptor agonist* OR anti-endotoxin compound* OR blocking complement* OR activated vitamin C OR mesenchymal stem cell* OR immune checkpoint inhibitor* OR extracorporeal blood removal OR corticosteroid*):ti,ab,kw 67451

#3 (precision medicine OR immunosuppress* OR anti-nflammatory agent* OR anticoagulant* OR cytokines OR granulocyte colon stimulating factor* OR granulocyte macrophage colony-stimulating factor OR immunoglobulin* OR programmed cell death 1 receptor* OR Toll-Like Receptors OR polymyxin B OR thymosin OR protein OR anti-infective agent* OR glucocorticoid* OR hydroxycorticosteroid* OR mesenchymal stem cell*):ti,ab,kw 126191

#4 #2 or #3 171251

#5 #1 and #4 in Trials 3246

ClinicaTrials.gov
2021 May 04
1309 Studies found for: Interventional Studies | sepsis | Adult, Older Adult

EMBASE (Ovid):
Database(s): Embase Classic+Embase 1947 to 2021 May 03

Slim MA, *et al. BMJ Open* 2022; 12:e060411. doi: 10.1136/bmjopen-2021-060411

Supplementary Table 1. Search Strategy EMBASE:

#	Searches	Results
**1**	exp *sepsis/dt, th or (sepsis or septic).ti,ab,kw.	233069
**2**	exp immunotherapy/ or personalized medicine/ or exp immunosuppressive treatment/	497119
**3**	exp antiinflammatory agent/dt, th or exp antiinfective agent/dt, th or drotrecogin/ or exp corticosteroid/dt, th or exp anticoagulant agent/dt, th or exp cytokine/dt or granulocyte colony stimulating factor/dt or granulocyte macrophage colony stimulating factor/dt or exp immunoglobulin/dt, th or programmed death 1 receptor/dt or toll like receptor/dt or polymyxin B/ or thymosin/dt or exp mesenchymal stem cell/	1908414
**4**	((immunotherap* or interleukin* or interferon or toll-like-receptor agonist* or anti-endotoxin compound* or blocking complement* or activated vitamin C or mesenchymal stem cell* or immune checkpoint inhibitor* or extracorporeal blood removal or corticosteroid*) and (treat* or therap*)).ti,ab,kw.	532386
**5**	2 or 3 or 4	2556542
**6**	exp clinical trial/ or controlled clinical trial/ or controlled study/ or randomized controlled trial/	8934963
**7**	((randomized or randomised or controlled) adj3 trial*).ti,ab,kw.	623934
**8**	clinical trial*.ti,ab,kw.	600136
**9**	6 or 7 or 8	9356625
**10**	1 and 5 and 9	18560
**11**	(exp child/ or exp pediatrics/ or (child* or infan* or newborn* or neonat* or baby or babies or pediatr* or paediatr*).ti,ab.) not adult/	3086878
**12**	10 not 11	15898
**13**	(exp animal/ or exp animal experiment/ or exp animal model/ or exp veterinary medicine/ or (animal* or monkey* or sheep or ovine or lamb or lambs or goat* or pig or pigs or swine or porcine or pup or pups or dog or dogs or canine or bitch* or beagle or feline or rodent* or rabbit* or rat or rats or mice or mouse or murine or cow or cows or horse or horses or ape or apes or gorilla or gorillas).ti,ab,kw.) not human/	6558145
**14**	12 not 13	12416
**15**	letter/ or editorial/ or note/ or case report/ or conference paper/ or "review"/ or (letter or comment* or editorial or case report).ti.	8552442
**16**	14 not 15	9437

Supplementary Table 2. Data Abstraction Table – Randomized controlled trials – "Targeting (excessive) inflammation"

	Innate immune response	Complement	Immunomodulation / endothelial dysfunctions	Immunomodulation by pleiotropic drugs	Immunonutrition	Immunomodulation by supportive treatments	Non-pharmalogical immunomodulatory strategies
**Author(s)**							
**Year**							
**Location**							
**Aim(s)**							
**Population**							
**Number of patients**							
**Design**							
**Intervention (dose, frequency, duration)**							
**Comparator (if any)**							
**Analyses (crude and adjusted)**							
**Main findings**							
**Measured immune profiles**							
**Personalized medicine; If so, how?**							
**Limitations as stated by author**							
**Quality of evidence**							

Supplementary Table 3. Data Abstraction Table – Randomized controlled trials – "Immune stimulation"

	Immunostimulatory cytokines and growth factors	Intravenous immunoglobulins	Mesenchymal stem cells	Immune checkpoint inhibitors
**Author(s)**				
**Year**				
**Location**				
**Aim(s)**				
**Population**				
**Number of patients**				
**Design**				
**Intervention (dose, frequency, duration)**				
**Comparator (if any)**				
**Analyses (crude and adjusted)**				
**Main findings**				
**Measured immune profiles**				
**Personalized medicine; If so, how?**				
**Limitations as stated by author**				
**Quality of evidence**				

Slim MA, *et al. BMJ Open* 2022; 12:e060411. doi: 10.1136/bmjopen-2021-060411

Supplementary Table 4. Data Abstraction Table – Observational controlled studies – "Targeting (excessive) inflammation"

	Innate immune response	Complement	Immunomodulation / endothelial dysfunctions	Immunomodulation by pleiotropic drugs	Immunonutrition	Immunomodulation by supportive treatments	Non-pharmalogical immunomodulatory strategies
**Author(s)**							
**Year**							
**Location**							
**Aim(s)**							
**Population**							
**Number of patients**							
**Design**							
**Intervention (dose, frequency, duration)**							
**Comparator (if any)**							
**Analyses (crude and adjusted)**							
**Main findings**							
**Measured immune profiles**							
**Personalized medicine; If so, how?**							
**Limitations as stated by author**							
**Quality of evidence**							

Supplementary Table 5. Data Abstraction Table – Observational controlled studies – "Immune stimulation"

	Immunostimulatory cytokines and growth factors	Intravenous immunoglobulins	Mesenchymal stem cells	Immune checkpoint inhibitors
Author(s)				
Year				
Location				
Aim(s)				
Population				
Number of patients				
Design				
Intervention (dose, frequency, duration)				
Comparator (if any)				
Analyses (crude and adjusted)				
Main findings				
Measured immune profiles				
Personalized medicine; If so, how?				
Limitations as stated by author				
Quality of evidence				

Supplementary Table 6. Data Abstraction Table – trials currently recruiting or being studied

	Trial
**Investigator(s)**	
**Start Date**	
**Location**	
**Aim(s)**	
**Outcome(s)**	
**Population**	
**Number of patients**	
**Design**	
**Intervention (dose, frequency, duration)**	
**Comparator (if any)**	
**Measured immune profiles**	
**Personalized medicine; If so, how?**	

Slim MA, *et al. BMJ Open* 2022; 12:e060411. doi: 10.1136/bmjopen-2021-060411
