# Supplementary material for: Personalised immunotherapy in sepsis: a scoping review protocol
Source: BMJ Open. 2022 May 9;12(5):e060411. doi: 10.1136/bmjopen-2021-060411 (PMC9086601; doi:10.1136/bmjopen-2021-060411)
Supplement: Author's manuscript [file bmjopen-2021-060411.draft_revisions.pdf]

BMJ Open is committed to open peer review. As part of this commitment we make the peer review history of every article we publish publicly available.

When an article is published we post the peer reviewers' comments and the authors' responses online. We also post the versions of the paper that were used during peer review. These are the versions that the peer review comments apply to.

The versions of the paper that follow are the versions that were submitted during the peer review process. They are not the versions of record or the final published versions. They should not be cited or distributed as the published version of this manuscript.

BMJ Open is an open access journal and the full, final, typeset and author-corrected version of record of the manuscript is available on our site with no access controls, subscription charges or pay-per-view fees (http://bmjopen.bmj.com).

If you have any questions on BMJ Open's open peer review process please email info.bmjopen@bmj.com

BMJ Open

Personalized immunotherapy in sepsis: a scoping review protocol

Journal:	*BMJ Open*
Manuscript ID	bmjopen-2021-060411
Article Type:	Protocol
Date Submitted by the Author:	20-Dec-2021

Complete List of Authors:	Slim, Marleen; Amsterdam UMC Location AMC, Intensive Care; Amsterdam UMC Location AMC, Center for Experimental and Molecular Medicine<br>van Mourik, Niels; Amsterdam UMC Location AMC, Intensive Care; Amsterdam UMC Location AMC, Center for Experimental and Molecular Medicine<br>Dionne, Joanna; McMaster University, Medicine<br>Oczkowski, Simon; McMaster University, Medicine<br>Netea, M; Radboudumc, Internal Medicine ; Radboudumc, Radboud Center for Infectious Diseases<br>Pickkers, Peter; Radboudumc, Intensive Care<br>Giamarellos-Bourboulis, Evangelos; National and Kapodistrian University of Athens, 4th Department of Internal Medicine<br>Müller, Marcella; Amsterdam UMC Location AMC, Intensive Care<br>van der Poll, Tom; Amsterdam UMC Location AMC, Internal Medicine, Division of Infectious Diseases; Amsterdam University Medical Centres, Center of Experimental and Molecular Medicine<br>Wiersinga, Joost; Amsterdam UMC Location AMC, Internal Medicine, Division of Infectious Diseases; Amsterdam UMC Location AMC, Center for Experimental and Molecular Medicine<br>Vlaar, Alexander; Amsterdam UMC Location AMC, Intensive Care<br>van Vught, Lonneke ; Amsterdam UMC Location AMC, Intensive Care; Amsterdam UMC Location AMC, Center for Experimental and Molecular Medicine

Keywords:	INFECTIOUS DISEASES, Adult intensive & critical care < INTENSIVE & CRITICAL CARE, Immunology < NATURAL SCIENCE DISCIPLINES, Clinical trials < THERAPEUTICS

SCHOLARONE™
Manuscripts

Personalized immunotherapy in sepsis: a scoping review protocol

M.A. Slim[1,2]*, N. van Mourik[1,2]*, J.C. Dionne[3,4,5,6], S.J.W. Oczkowski[3,4,5], M.G. Netea[7], P. Pickkers[8], E. J. Giamarellos-Bourboulis[9], M.C.A. Müller[2], T. van der Poll[1,10], W.J. Wiersinga[1,10], A.P.J. Vlaar[2], L.A. van Vught[1,2]

* Both authors contributed equally.

Affiliations

[1] Center for Experimental and Molecular Medicine, Amsterdam University Medical, University of Amsterdam, Amsterdam, The Netherlands

[2] Department of Intensive Care, Amsterdam University Medical Centers, University of Amsterdam, Amsterdam, The Netherlands

[3] Department of Medicine, McMaster University, Hamilton, Canada.

[4] The Guidelines in Intensive Care Development and Evaluation (GUIDE) Group, Research Institute St. Joseph's Healthcare Hamilton, Hamilton, Canada.

[5] Department of Health Research Methods, Evidence, and Impact, McMaster University, Hamilton, Canada.

[6] Division of Gastroenterology, McMaster University, Hamilton, ON, Canada.

[7] Department of Internal Medicine and Radboud Center for Infectious Diseases, Radboud University Medical Center, Nijmegen, The Netherlands

[8] Department of Intensive Care Medicine, Radboud University Medical Center

[9] 4th Department of Internal Medicine, National and Kapodistrian University of Athens, Medical School, Athens, Greece

[10] Department of Internal Medicine, Division of Infectious Diseases, Amsterdam University Medical Centers, University of Amsterdam, Amsterdam, The Netherlands

Corresponding author

Marleen Slim; Department of Intensive Care Medicine, Amsterdam University Medical Center, Meibergdreef 9, Room G3-220, 1105 AZ Amsterdam, the Netherlands. E: m.a.slim@amsterdamumc.nl

Manuscript details

Journal:	BMJ Open
Article type:	Protocol
Word count abstract:	288 (out of 300)
Word count article:	1680 (out of 4000)
Table count:	4 (all in the supplement)
Reference count:	20

For peer review only

Abstract

Introduction. Sepsis, a life-threatening organ dysfunction syndrome occurring in the context of severe infections, remains a major burden on global health with high morbidity and high mortality rates. Despite recent advances in the understanding of its pathophysiology, the treatment of sepsis remains supportive of nature with few interventions specifically designed for treating this complex syndrome. The focus of sepsis trials has increasingly shifted towards targeting excessive inflammation and immunosuppression using immunomodulatory agents. However, it remains uncertain how to identify patients that could benefit from such treatment, whether treatments can be tailored to an individual's immune profile, or at which stage of the disease the intervention should be initiated. In this scoping review, we provide a comprehensive overview of current available literature on immunostimulatory and immunosuppressive therapies against sepsis.

Methods and analysis. The aim of this scoping review is to describe and summarize current literature evaluating immunotherapy in adult patients with sepsis. The review will be performed using the framework formulated by Arksey and O'Malley. A comprehensive literature and study collection will be executed by searching PubMed, Embase, Cochrane CENTRAL and ClinicalTrials.gov to identify clinical trials and cohort studies concerning immunotherapy in adult patients with sepsis. Screening will be performed independently and in duplicate by two reviewers who will also independently extract data into pre-specified spreadsheets. We will summarize evidence in tabular format with descriptive statistics. The reported evidence will convey knowledge on the types of immunotherapies studied, and currently being studied, in adult patients with sepsis.

Ethics and dissemination. Approval from a medical ethics committee is not required. Once completed, the review will be submitted for publication in a peer-reviewed journal. These results will be of value to clinicians and researchers with an interest in advancing sepsis care.

For peer review only - http://bmjopen.bmj.com/site/about/guidelines.xhtml

Article Summary

Strengths and limitations of this study

- This scoping review will summarizing which immunotherapies have been, and are currently being, studied; highlighting the gaps in in the field of immunotherapy in sepsis.
- As this will be a scoping review, the broad topic of immunotherapy in sepsis, in which many different study designs might be applicable, can be addressed in a complete and structured manner.
- Compared to previous reviews on this topic, which mainly describe current advances in a narrative way, we here plan to use a systematic approach to identify and summarize the evidence for immunomodulatory agents.
- This review will focus on studies that used a personalized approach to immunotherapy, which is proposed to be the future of sepsis research.
- A potential limitation of this review lies within the heterogeneity between trials; sepsis is a heterogeneous syndrome and trials use trial-specific in-and exclusion criteria, leading to possible heterogeneity within the population studied.

For peer review only

For peer review only - http://bmjopen.bmj.com/site/about/guidelines.xhtml

Introduction

Rationale

Sepsis is defined as a life-threatening organ dysfunction syndrome caused by a dysregulated host response to an infection[1 2]. The global disease burden of sepsis remains high, with 48.9 million cases annually and almost 20% of all global deaths are sepsis-related[3]. Over the past few decades, significant progress in the understanding of the pathophysiology of sepsis has been made. Early diagnosis, timely antibiotic treatment and organ support are pivotal and are associated with a decrease in mortality rates[2]. Despite numerous efforts to date, there are few therapies which target the immunological basis of this life-threatening condition[4]. So far, treatment of sepsis mainly revolves around supportive care; administering antibiotics, fluids and vasopressors or initiating organ support.

During sepsis, the host response may be altered in multiple ways, explaining the highly heterogeneous clinical presentation, treatment responses and prognosis[5]. While the main pathophysiological cause of sepsis is often thought to be an excessive inflammatory immune response to an infection, it is now understood that a secondary immunosuppressive response often occurs concurrently[4-6]. Modulating the immune response to infection represents a promising treatment target in sepsis. However, over 100 clinical trials investigating the use of immunomodulatory agents in sepsis have been unsuccessful in demonstrating a benefit of immunomodulatory therapy[4 7]. One reason these trials may not have demonstrated a benefit is the use of a “one-size-fits-all” approach to immunotherapy. Sepsis is a clinically heterogeneous disease syndrome, with individual patients demonstrating varied clinical responses to infections. This suggests that personalized immunomodulatory treatment, tailored to an individual patient’s immune profile, may be a better approach to treating patients suffering from sepsis[8]. Trials using personalized immunotherapy could increase the number of patients in the study with the potential to benefit from the administered treatment, and minimize the number of patients exposed to treatments which are unlikely to help or even cause harm[8]. The first step towards implementation of personalized medicine using immunomodulatory treatment is to provide a

For peer review only - http://bmjopen.bmj.com/site/about/guidelines.xhtml

structured and in-depth overview of currently available evidence on immunotherapy in the treatment of sepsis.

Study aim

The purpose of this scoping review is to describe and summarize current literature evaluating immunotherapy in adult patients with sepsis and the extent to which these trials used a personalized medicine approach.

Methods and analysis

The present study will be a scoping review performed using previously defined methods[9 10]. The model by Arksey and O'Malley defines a five-stage methodological framework, which includes; (1) identifying the research question, (2) identifying relevant studies, (3) study selection, (4) charting the data, and (5) collating, summarizing and reporting the results[9].

Stage 1: Identifying the research question

The following research questions will be addressed:

Primary objectives

1. Which immunotherapies have been, or are currently being, studied in adult patients with sepsis?
2. What are the study characteristics and patient populations of trials on immunotherapy in adult patients with sepsis?
3. How have clinical trials and cohort studies on sepsis used a personalized immunotherapy approach (including clinical phenotypes; immunoprofiling: cell populations, protein biomarkers and/or immune assays), and have these approaches changed over time?

Stage 2: Identifying relevant studies

For peer review only - http://bmjopen.bmj.com/site/about/guidelines.xhtml

A comprehensive literature search will be conducted using PubMed, Embase, Cochrane CENTRAL and ClinicalTrials.gov. Inclusion criteria are 1) clinical trials or cohort studies (including case control studies and observational cohorts) 2) investigating immunomodulatory therapies in 3) adult (≥ 16 years) patients with sepsis. All studies addressing therapies with an established immunomodulatory effect will be included. Studies addressing therapies with a hypothesized immunomodulatory effect (e.g. antibiotics, fluids, statins, immunonutrition, β-blockers, vasopressors) will be included, but only if the study investigates the immunomodulatory effects of the treatment. After completion of the initial literature search, we will use a snowball approach to find additional studies of interest[11]. All articles must contain full text written in English or Dutch. Exclusion criteria are 1) case studies, 2) animal studies and 3) studies in healthy volunteers. Systematic reviews and meta-analyses will also be excluded, but searched for potentially relevant references.

Search keywords include sepsis, immunotherapy, precision medicine, immunosuppression, anti-inflammatory agents, corticosteroid, anticoagulants, cytokines, granulocyte colony-stimulating factor, immunoglobulins, programmed cell death 1 receptor, Toll-like receptors, Polymyxin B, thymosin, protein C, mesenchymal stem cells, anti-endotoxin compound and extracorporeal blood removal. Articles retrieved from the database searches will be imported into Rayyan[12], an open-source program, to organize and facilitate screening of the search results. The full search strategies can be found in the **Supplementary Methods and Supplementary Table 1**. Prior to publication the search will be repeated to enable the inclusion of recently published studies.

Stage 3: Study selection

We will approach the study selection systematically using the inclusion and exclusion criteria described above, providing consistency in the decision-making regarding article selection. Screening will be performed independently and in duplicate by two reviewers (MS, NvM). The first step will be to screen all included articles in Rayyan on title and abstract. Disagreements at the trial/abstract stage will be

For peer review only - http://bmjopen.bmj.com/site/about/guidelines.xhtml

resolved by discussion between the two reviewers. When uncertainty or disagreement remains, a third reviewer (LvV) will be consulted to make a final decision regarding inclusion of the study. Final inclusion will be determined based on the analysis of the full text of any papers included by either reviewer during title/abstract screening stage. Disagreements at the full-text stage will be resolved in the same manner as described above at the title/abstract stage.

Stage 4: Charting the data

Data to be charted include:

1. Author(s), year of publication, study location;
2. Aim(s) of the study;
3. Study population, in- and exclusion criteria, number of patients;
4. Study design;
5. The type of immunotherapy and comparator (if any) used;
6. Main findings and outcome measures, including measured immune profiles;
7. If a personalized approach was used, and if so, which approach;
8. Limitations of the study stated by its authors;
9. Quality of evidence.

The quality of evidence will be critically appraised using the RoB 2 tool (a revised Cochrane risk of bias tool for randomized trials)[13] and the Newcastle Ottawa Quality Assessment Scale for Cohort Studies[14].

Two reviewers (MS, NvM) will independently extract data into pre-specified spreadsheets (**Supplementary Table 2 and 3**). At regular intervals, extracted data from the included articles in this framework will be compared in order to ensure interrater reliability. Any disagreements or inconsistencies will be resolved by discussion, or, when uncertainty or disagreement remains, a third reviewer (LvV) will be consulted.

For peer review only - http://bmjopen.bmj.com/site/about/guidelines.xhtml

Stage 5: collating, summarizing and reporting the results

We will present an overview of the reviewed literature in tabular format with descriptive statistics. In addition, when possible, we will analyze treatment groups including (but not restricted to) strategies that modulate excessive inflammation (treatments targeting the innate immune response, complement, immunothrombosis and endothelial dysfunction, pleiotropic drugs with immunomodulatory effects, immunonutrition, supportive treatments with immunomodulatory effects, and non-pharmalogical immunomodulatory strategies) and strategies aiming at immune stimulation (immunomodulatory cytokines and growth factors, intravenous immunoglobulins, mesenchymal stem cells, and immune checkpoint inhibitors). Furthermore, we will present an overview of the trials currently recruiting or being studied in a supplementary table (**Supplementary Table 4**).

Patient and public involvement

No patient and/or public involved.

Ethics and dissemination

As this is scoping review, approval from a medical ethics committee is not required. Once completed, the review will be submitted for publication in a peer-reviewed journal.

Discussion

This scoping review will provide structured and detailed information on the types of immunotherapies studied in adult patients with sepsis. It will provide insight into how immunomodulatory trials have been conducted, and to what extent a personalized approach to immunotherapy has been used. An overview of potential immunostimulatory and immunosuppressive treatments will be given,

For peer review only - http://bmjopen.bmj.com/site/about/guidelines.xhtml

For peer review only

highlighting gaps in the field of sepsis treatment, and identifying strategies to study personalized immunotherapies in adult patients with sepsis.

In this review we will provide background on pathophysiological processes targeted within the investigated therapies. Compared to previous reviews on this topic[5 7 15-18], which mainly describe current advances in a narrative way, we here use a systematic approach to identify and summarize the evidence for immunomodulatory agents. In addition, the current review will add to the field of sepsis treatment by focusing on the evidence of personalized therapy.

This review will have several methodological strengths. First, as this will be a scoping review, the broad topic of immunotherapy in sepsis in which many different study designs might be applicable can be addressed in a complete and structured manner[9]. Second, we will systematically assess the risk of systematic errors via bias risk assessments, which will provide robustness of our results and conclusions. Third, we will focus on studies that used a personalized approach to immunotherapy, which is proposed to be the future of sepsis research[19 20].

A potential limitation of this review lies within the heterogeneity between trials. However, this is inherent when studying clinical trials with strict in- and exclusion criteria and is probably a good reflection of daily clinical practice in treating sepsis patients. Although inevitable, different criteria for sepsis have been used over time[2], leading to heterogeneity in the population included.

In conclusion, finding suitable treatment options for sepsis is crucial for improving the patient's outcome. Summarizing which immunotherapies have been, and are currently being, studied will highlight the gaps in in the field of immunotherapy in sepsis. This will be essential to improve further knowledge in the treatment of sepsis.

For peer review only - http://bmjopen.bmj.com/site/about/guidelines.xhtml

Author contributions

MS, TvdP, WJW, AV and LvV formed the study and outlined the proposal. This protocol, including the supplement, was written by MS, NM and LvV. MS, NvM, JD, SO, MM, AV and LvV have made a substantive intellectual contribution to the design of the protocol, the study aim, and research questions. They jointly developed the search strategy and data extraction framework. All authors edited the manuscript and approved the final version.

Funding statement

MS, NvM, MN, PP, EGB, TvdP, WJW and AV are supported by the European Commission (Horizon 2020, ImmunoSep, grant number 847422). WJW is supported by the Netherlands Organization for Scientific Research (Nederlandse Organisatie voor Wetenschappelijk Onderzoek; VIDI grant 91716475). LvV is supported by the Netherlands Organization for Scientific Research (VENI grant 09150161910033). The funding sources had no involvement in the study design, in the writing of the protocol and in the decision to submit the article for publication.

Competing interests statement

SO reports grants and/or contracts from Physician Services Incorporated Ontario and Canadian Institutes of Health Research, and support from the American Thoracic Society and the European Respiratory Society. MN reports an ERC Advanced Grant, a Spinoza Grant and is on the scientific advisory board of SOBI. PP is on the DSMB of the IL-7 trial 'ILEAD'. EGB reports grants and/or contracts from Abbott CH, bioMerieux, MSD, Sobi, and ThermoFisher BRAHMS. MM is on the DSMB of the PACER trial and the National Dutch Guideline on anticoagulation in COVID-19. TvdP reports project grants from the European Commission and is on the DSMBs of RECAP-CAP, SuDDICU and the CONFIDENT trial. WJW reports a project grant from ZonMW/NWO and receives consulting fees from Merck, Sobi and GlaxoSmithKline. AV receives consulting fees from InflaRx. The other authors have nothing to declare.

For peer review only - http://bmjopen.bmj.com/site/about/guidelines.xhtml

References

1. Seymour CW, Liu VX, Iwashyna TJ, et al. Assessment of Clinical Criteria for Sepsis: For the Third International Consensus Definitions for Sepsis and Septic Shock (Sepsis-3). *JAMA* 2016;315(8):762-74. doi: 10.1001/jama.2016.0288
2. Singer M, Deutschman CS, Seymour CW, et al. The Third International Consensus Definitions for Sepsis and Septic Shock (Sepsis-3). *JAMA* 2016;315(8):801-10. doi: 10.1001/jama.2016.0287
3. Rudd KE, Johnson SC, Agesa KM, et al. Global, regional, and national sepsis incidence and mortality, 1990–2017: analysis for the Global Burden of Disease Study. *The Lancet* 2020;395(10219):200-11. doi: 10.1016/s0140-6736(19)32989-7
4. van der Poll T, van de Veerdonk FL, Scicluna BP, et al. The immunopathology of sepsis and potential therapeutic targets. *Nat Rev Immunol* 2017;17(7):407-20. doi: 10.1038/nri.2017.36
5. Steinhagen F, Schmidt SV, Schewe JC, et al. Immunotherapy in sepsis - brake or accelerate? *Pharmacol Ther* 2020;208:107476. doi: 10.1016/j.pharmthera.2020.107476
6. Rubio I, Osuchowski MF, Shankar-Hari M, et al. Current gaps in sepsis immunology: new opportunities for translational research. *The Lancet Infectious Diseases* 2019;19(12):e422-e36. doi: 10.1016/s1473-3099(19)30567-5
7. Davies R, O'Dea K, Gordon A. Immune therapy in sepsis: Are we ready to try again? *J Intensive Care Soc* 2018;19(4):326-44. doi: 10.1177/1751143718765407
8. Marshall JC. Why have clinical trials in sepsis failed? *Trends Mol Med* 2014;20(4):195-203. doi: 10.1016/j.molmed.2014.01.007
9. Arksey H, O'Malley L. Scoping studies: towards a methodological framework. *International Journal of Social Research Methodology* 2005;8(1):19-32. doi: 10.1080/1364557032000119616
10. JBI Scoping Review Network - Resources [Available from: https://jbi.global/scoping-review-network/resources] Accessed October 18, 2021.
11. Wohlin C. Guidelines for snowballing in systematic literature studies and a replication in software engineering. Proceedings of the 18th International Conference on Evaluation and Assessment in Software Engineering - EASE '14, 2014:1-10.
12. Ouzzani M, Hammady H, Fedorowicz Z, et al. Rayyan-a web and mobile app for systematic reviews. *Syst Rev* 2016;5(1):210. doi: 10.1186/s13643-016-0384-4
13. Risk of bias tool - RoB 2 tool [Available from: https://sites.google.com/site/riskofbiastool/welcome/rob-2-0-tool?authuser=0] Accessed October 18, 2021.

For peer review only - http://bmjopen.bmj.com/site/about/guidelines.xhtml

14. Newcastle - Ottawa Quality Assessment Scale - Cohort Studies [Available from: https://www.ncbi.nlm.nih.gov/books/NBK115843/bin/appe-fm3.pdf] Accessed on October 18, 2021.

15. Hotchkiss RS, Monneret G, Payen D. Sepsis-induced immunosuppression: from cellular dysfunctions to immunotherapy. *Nat Rev Immunol* 2013;13(12):862-74. doi: 10.1038/nri3552

16. Hotchkiss RS, Monneret G, Payen D. Immunosuppression in sepsis: a novel understanding of the disorder and a new therapeutic approach. *The Lancet Infectious Diseases* 2013;13(3):260-68. doi: 10.1016/s1473-3099(13)70001-x

17. Patil NK, Bohannon JK, Sherwood ER. Immunotherapy: A promising approach to reverse sepsis-induced immunosuppression. *Pharmacol Res* 2016;111:688-702. doi: 10.1016/j.phrs.2016.07.019

18. van der Poll T. Immunotherapy of sepsis. *The Lancet Infectious Diseases* 2001;1(3):165-74. doi: 10.1016/s1473-3099(01)00093-7

19. Rello J, Valenzuela-Sanchez F, Ruiz-Rodriguez M, et al. Sepsis: A Review of Advances in Management. *Adv Ther* 2017;34(11):2393-411. doi: 10.1007/s12325-017-0622-8

20. Rello J, van Engelen TSR, Alp E, et al. Towards precision medicine in sepsis: a position paper from the European Society of Clinical Microbiology and Infectious Diseases. *Clin Microbiol Infect* 2018;24(12):1264-72. doi: 10.1016/j.cmi.2018.03.011

For peer review only

For peer review only - http://bmjopen.bmj.com/site/about/guidelines.xhtml

Supplementary online content

Personalized immunotherapy in sepsis: a scoping review protocol

M.A. Slim*, N. van Mourik*, J.C. Dionne, S.J.W. Oczkowski, M.G. Netea, P. Pickkers, E. J. Giamarellos-Bourboulis, M.C.A. Müller, T. van der Poll, W.J. Wiersinga, A.P.J. Vlaar, L.A. van Vught
* Both authors contributed equally.

Content	Page
Supplementary Methods	2
Supplementary Table 1.	4
Supplementary Table 2.	5
Supplementary Table 3.	6
Supplementary Table 4.	7

For peer review only

Supplementary Methods. The full search string

PubMed
2021 May 04

2444 hits:
("Sepsis"[Majr] OR sepsis[tiab] OR septic[tiab])

AND

("Immunotherapy"[Mesh] OR "Precision Medicine"[Mesh] OR "Immunosuppression"[Mesh] OR "Anti-Inflammatory Agents/therapeutic use"[Mesh] OR "Anti-Infective Agents/therapeutic use"[Mesh] OR "Glucocorticoids"[Mesh] OR "Hydroxycorticosteroids"[Mesh] OR "Anticoagulants/therapeutic use"[Mesh] OR "Cytokines/antagonists and inhibitors"[Mesh] OR "Cytokines/therapeutic use"[Mesh] OR "Granulocyte Colony-Stimulating Factor/therapeutic use"[Mesh] OR "Granulocyte-Macrophage Colony-Stimulating Factor/therapeutic use"[Mesh] OR "Immunoglobulins, Intravenous/therapeutic use"[Mesh] OR "Programmed Cell Death 1 Receptor/therapeutic use"[Mesh] OR "Toll-Like Receptors/therapeutic use"[Mesh] OR "Polymyxin B/therapeutic use"[Mesh] OR "Thymosin/therapeutic use"[Mesh] OR "Protein C/therapeutic use"[Mesh] OR "Mesenchymal Stem Cells"[Mesh] OR immunotherap*[tiab] OR interleukin*[tiab] OR interferon[tiab] OR toll-like-receptor agonist*[tiab] OR anti-endotoxin compound*[tiab] OR blocking complement*[tiab] OR activated vitamin C[tiab] OR mesenchymal stem cell*[tiab] OR immune checkpoint inhibitor*[tiab] OR extracorporeal blood removal[tiab] OR corticosteroid*[tiab])

AND

("Clinical Trial" [Publication Type] OR clinical trial*[tiab] OR randomized trial*[tiab] OR randomized trial*[tiab] OR controlled trial*[tiab])

NOT

(("Child"[Mesh] OR "Infant"[Mesh] OR "Pediatrics"[Mesh] OR child*[tiab] OR infan*[tiab] OR newborn*[tiab] OR neonat*[tiab] OR baby[tiab] OR babies[tiab] OR pediatr*[tiab] OR paediatr*[tiab]) NOT "Adult"[Mesh])

NOT

("Editorial"[Publication Type] OR "Letter"[Publication Type] OR "News"[Publication Type] OR "Comment"[Publication Type] OR "Review" [Publication Type] OR "Case Reports"[Publication Type] OR "letter*"[Title] OR "comment*"[Title] OR editorial[ti])

NOT

(("Animals"[MeSH Terms] OR "Animal Experimentation"[MeSH Terms] OR "animals, laboratory"[MeSH Terms] OR "models, animal"[MeSH Terms] OR "animal*"[Title/Abstract] OR "rat"[Title/Abstract] OR

For peer review only - http://bmjopen.bmj.com/site/about/guidelines.xhtml

"rats"[Title/Abstract] OR "mice"[Title/Abstract] OR "mouse"[Title/Abstract] OR "dog"[Title/Abstract] OR "dogs"[Title/Abstract] OR "pig"[Title/Abstract] OR "pigs"[Title/Abstract] OR "cow"[Title/Abstract] OR "cows"[Title/Abstract] OR "monkey"[Title/Abstract] OR "monkeys"[Title/Abstract] OR "horse"[Title/Abstract] OR "horses"[Title/Abstract] OR "sheep"[Title/Abstract] OR "ovine"[Title/Abstract] OR "lamb"[Title/Abstract] OR "lambs"[Title/Abstract] OR "goat*"[Title/Abstract] OR "swine"[Title/Abstract] OR "porcine"[Title/Abstract] OR "pup"[Title/Abstract] OR "pups"[Title/Abstract] OR "canine"[Title/Abstract] OR "bitch*"[Title/Abstract] OR "beagle"[Title/Abstract] OR "feline"[Title/Abstract] OR "rodent*"[Title/Abstract] OR "rabbit*"[Title/Abstract] OR "murine"[Title/Abstract] OR "ape"[Title/Abstract] OR "apes"[Title/Abstract] OR "gorilla"[Title/Abstract] OR "gorillas"[Title/Abstract] OR "catfish"[Title/Abstract]) NOT "Humans"[MeSH Terms])

Cochrane Central Register of Controlled Trials

Issue 4 of 12, April 2021

ID Search Hits

#1 (sepsis or septic):ti,ab,kw 13698

#2 (immunotherap* OR interleukin* OR interferon OR toll-like-receptor agonist* OR anti-endotoxin compound* OR blocking complement* OR activated vitamin C OR mesenchymal stem cell* OR immune checkpoint inhibitor* OR extracorporeal blood removal OR corticosteroid*):ti,ab,kw 67451

#3 (precision medicine OR immunosuppress* OR anti-nflammatory agent* OR anticoagulant* OR cytokines OR granulocyte colon stimulating factor* OR granulocyte macrophage colony-stimulating factor OR immunoglobulin* OR programmed cell death 1 receptor* OR Toll-Like Receptors OR polymyxin B OR thymosin OR protein OR anti-infective agent* OR glucocorticoid* OR hydroxycorticosteroid* OR mesenchymal stem cell*):ti,ab,kw 126191

#4 #2 or #3 171251

#5 #1 and #4 in Trials 3246

ClinicaTrials.gov

2021 May 04

1309 Studies found for: Interventional Studies | sepsis | Adult, Older Adult

EMBASE (Ovid):

Database(s): Embase Classic+Embase 1947 to 2021 May 03

For peer review only - http://bmjopen.bmj.com/site/about/guidelines.xhtml

Supplementary Table 1. Search Strategy EMBASE:

#	Searches	Results
**1**	exp *sepsis/dt, th or (sepsis or septic).ti,ab,kw.	233069
**2**	exp immunotherapy/ or personalized medicine/ or exp immunosuppressive treatment/	497119
**3**	exp antiinflammatory agent/dt, th or exp antiinfective agent/dt, th or drotrecogin/ or exp corticosteroid/dt, th or exp anticoagulant agent/dt, th or exp cytokine/dt or granulocyte colony stimulating factor/dt or granulocyte macrophage colony stimulating factor/dt or exp immunoglobulin/dt, th or programmed death 1 receptor/dt or toll like receptor/dt or polymyxin B/ or thymosin/dt or exp mesenchymal stem cell/	1908414
**4**	((immunotherap* or interleukin* or interferon or toll-like-receptor agonist* or anti-endotoxin compound* or blocking complement* or activated vitamin C or mesenchymal stem cell* or immune checkpoint inhibitor* or extracorporeal blood removal or corticosteroid*) and (treat* or therap*)).ti,ab,kw.	532386
**5**	2 or 3 or 4	2556542
**6**	exp clinical trial/ or controlled clinical trial/ or controlled study/ or randomized controlled trial/	8934963
**7**	((randomized or randomised or controlled) adj3 trial*).ti,ab,kw.	623934
**8**	clinical trial*.ti,ab,kw.	600136
**9**	6 or 7 or 8	9356625
**10**	1 and 5 and 9	18560
**11**	(exp child/ or exp pediatrics/ or (child* or infan* or newborn* or neonat* or baby or babies or pediatr* or paediatr*).ti,ab.) not adult/	3086878
**12**	10 not 11	15898
**13**	(exp animal/ or exp animal experiment/ or exp animal model/ or exp veterinary medicine/ or (animal* or monkey* or sheep or ovine or lamb or lambs or goat* or pig or pigs or swine or porcine or pup or pups or dog or dogs or canine or bitch* or beagle or feline or rodent* or rabbit* or rat or rats or mice or mouse or murine or cow or cows or horse or horses or ape or apes or gorilla or gorillas).ti,ab,kw.) not human/	6558145
**14**	12 not 13	12416
**15**	letter/ or editorial/ or note/ or case report/ or conference paper/ or "review"/ or (letter or comment* or editorial or case report).ti.	8552442
**16**	14 not 15	9437

Supplementary Table 2. Data Abstraction Table – "Targeting (excessive) inflammation"

	Innate immune response	Complement	Immunomodulation / endothelial dysfunctions	Immunomodulation by pleiotropic drugs	Immunonutrition	Immunomodulation by supportive treatments	Non-pharmalogical immunomodulatory strategies
**Author(s)**							
**Year**							
**Location**							
**Aim(s)**							
**Population**							
**Number of patients**							
**Design**							
**Intervention (dose, frequency, duration)**							
**Comparator (if any)**							
**Analyses (crude and adjusted)**							
**Main findings**							
**Measured immune profiles**							
**Personalized medicine; If so, how?**							
**Limitations as stated by author**							
**Quality of evidence**							

Supplementary Table 3. Data Abstraction Table – "Immune stimulation"

	Immunostimulatory cytokines and growth factors	Intravenous immunoglobulins	Mesenchymal stem cells	Immune checkpoint inhibitors
Author(s)				
Year				
Location				
Aim(s)				
Population				
Number of patients				
Design				
Intervention (dose, frequency, duration)				
Comparator (if any)				
Analyses (crude and adjusted)				
Main findings				
Measured immune profiles				
Personalized medicine; If so, how?				
Limitations as stated by author				
Quality of evidence				

Supplementary Table 4. Data Abstraction Table – trials currently recruiting or being studied

	Trial
**Investigator(s)**	
**Start Date**	
**Location**	
**Aim(s)**	
**Outcome(s)**	
**Population**	
**Number of patients**	
**Design**	
**Intervention (dose, frequency, duration)**	
**Comparator (if any)**	
**Measured immune profiles**	
**Personalized medicine; If so, how?**	

er review only

PRISMA-P (Preferred Reporting Items for Systematic review and Meta-Analysis Protocols) 2015 checklist: recommended items to address in a systematic review protocol*

Section and topic	Item No	Checklist item	Information reported YES	NO		Page numbers
**ADMINISTRATIVE INFORMATION**						
Title:						
Identification	1a	Identify the report as a protocol of a systematic review	☒	☐		See page 1.
Update	1b	If the protocol is for an update of a previous systematic review, identify as such	☐	☒	N/A	
Registration	2	If registered, provide the name of the registry (such as PROSPERO) and registration number	☐	☒	This review could not be registered in the international prospective register of systematic reviews (PROSPERO) because they do not accept scoping reviews.	
Authors:						
Contact	3a	Provide name, institutional affiliation, e-mail address of all protocol authors; provide physical mailing address of corresponding author	☒	☐		See page 1.
Contributions	3b	Describe contributions of protocol authors and identify the guarantor of the review	☒	☐		See page 10.
Amendments	4	If the protocol represents an amendment of a previously completed or published protocol, identify as such and list changes; otherwise, state plan for documenting important protocol amendments	☐	☒	N/A	
Support:						
Sources	5a	Indicate sources of financial or other support for the review	☐	☒	There was no financial support for this review. For the financial support of the researchers see page 10.	
Sponsor	5b	Provide name for the review funder and/or sponsor	☐	☒	N/A	
Role of sponsor or funder	5c	Describe roles of funder(s), sponsor(s), and/or institution(s), if any, in developing the protocol	☐	☒	N/A	

1

For peer review only

**INTRODUCTION**						
Rationale	6	Describe the rationale for the review in the context of what is already known	☒	☐		See page 4.
Objectives	7	Provide an explicit statement of the question(s) the review will address with reference to participants, interventions, comparators, and outcomes (PICO)	☐	☒		
**METHODS**						
Eligibility criteria	8	Specify the study characteristics (such as PICO, study design, setting, time frame) and report characteristics (such as years considered, language, publication status) to be used as criteria for eligibility for the review	☒	☐		See page 6.
Information sources	9	Describe all intended information sources (such as electronic databases, contact with study authors, trial registers or other grey literature sources) with planned dates of coverage	☒	☐		See page 6, supplement pages 2-4.
Search strategy	10	Present draft of search strategy to be used for at least one electronic database, including planned limits, such that it could be repeated	☒	☐		See supplement pages 2-4.
Study records:						
Data management	11a	Describe the mechanism(s) that will be used to manage records and data throughout the review	☒	☐		See page 6.
Selection process	11b	State the process that will be used for selecting studies (such as two independent reviewers) through each phase of the review (that is, screening, eligibility and inclusion in meta-analysis)	☒	☐		See pages 6-7.
Data collection process	11c	Describe planned method of extracting data from reports (such as piloting forms, done independently, in duplicate), any processes for obtaining and confirming data from investigators	☒	☐		See pages 7-8.
Data items	12	List and define all variables for which data will be sought (such as PICO items, funding sources), any pre-planned data assumptions and simplifications	☒	☐		See supplement pages 5-7.
Outcomes and prioritization	13	List and define all outcomes for which data will be sought, including prioritization of main and additional outcomes, with rationale	☐	☒	Since this is a scoping review, all study specific outcomes will evaluated.	
Risk of bias in individual studies	14	Describe anticipated methods for assessing risk of bias of individual studies, including whether this will be done at the outcome or study level, or both; state how this information will be used in data synthesis	☒	☐		See page 7.
Data synthesis	15a	Describe criteria under which study data will be quantitatively synthesised	☐	☒		

For peer review only

	15b	If data are appropriate for quantitative synthesis, describe planned summary measures, methods of handling data and methods of combining data from studies, including any planned exploration of consistency (such as I^2, Kendall's τ)	☐	☒	
	15c	Describe any proposed additional analyses (such as sensitivity or subgroup analyses, meta-regression)	☐	☒	
	15d	If quantitative synthesis is not appropriate, describe the type of summary planned	☒	☐	See page 8.
Meta-bias(es)	16	Specify any planned assessment of meta-bias(es) (such as publication bias across studies, selective reporting within studies)	☐	☒	
Confidence in cumulative evidence	17	Describe how the strength of the body of evidence will be assessed (such as GRADE)	☐	☒	

*** It is strongly recommended that this checklist be read in conjunction with the PRISMA-P Explanation and Elaboration (cite when available) for important clarification on the items. Amendments to a review protocol should be tracked and dated. The copyright for PRISMA-P (including checklist) is held by the PRISMA-P Group and is distributed under a Creative Commons Attribution Licence 4.0.**

From: Shamseer L, Moher D, Clarke M, Ghersi D, Liberati A, Petticrew M, Shekelle P, Stewart L, PRISMA-P Group. Preferred reporting items for systematic review and meta-analysis protocols (PRISMA-P) 2015: elaboration and explanation. BMJ. 2015 Jan 2;349(jan02 1):g7647.

For peer review only

For peer review only - http://bmjopen.bmj.com/site/about/guidelines.xhtml

BMJ Open

Personalized immunotherapy in sepsis: a scoping review protocol

Journal:	*BMJ Open*
Manuscript ID	bmjopen-2021-060411.R1
Article Type:	Protocol
Date Submitted by the Author:	21-Apr-2022
Complete List of Authors:	Slim, Marleen; Amsterdam UMC Location AMC, Intensive Care; Amsterdam UMC Location AMC, Center for Experimental and Molecular Medicine<br>van Mourik, Niels; Amsterdam UMC Location AMC, Intensive Care; Amsterdam UMC Location AMC, Center for Experimental and Molecular Medicine<br>Dionne, Joanna; McMaster University, Medicine; Research Institute St. Joseph's Healthcare Hamilton, The Guidelines in Intensive Care Development and Evaluation (GUIDE) Group<br>Oczkowski, Simon; McMaster University, Medicine; Research Institute St. Joseph's Healthcare Hamilton, The Guidelines in Intensive Care Development and Evaluation (GUIDE) Group<br>Netea, M; Radboudumc, Internal Medicine ; Radboudumc, Radboud Center for Infectious Diseases<br>Pickkers, Peter; Radboudumc, Intensive Care<br>Giamarellos-Bourboulis, Evangelos; National and Kapodistrian University of Athens, 4th Department of Internal Medicine<br>Müller, Marcella; Amsterdam UMC Location AMC, Intensive Care<br>van der Poll, Tom; Amsterdam UMC Location AMC, Internal Medicine, Division of Infectious Diseases; Amsterdam University Medical Centres, Center of Experimental and Molecular Medicine<br>Wiersinga, Joost; Amsterdam UMC Location AMC, Internal Medicine, Division of Infectious Diseases; Amsterdam UMC Location AMC, Center for Experimental and Molecular Medicine<br>Vlaar, Alexander; Amsterdam UMC Location AMC, Intensive Care<br>van Vught, Lonneke ; Amsterdam UMC Location AMC, Intensive Care; Amsterdam UMC Location AMC, Center for Experimental and Molecular Medicine
**Primary Subject Heading**:	Infectious diseases
Secondary Subject Heading:	Intensive care, Pharmacology and therapeutics
Keywords:	INFECTIOUS DISEASES, Adult intensive & critical care < INTENSIVE & CRITICAL CARE, Immunology < NATURAL SCIENCE DISCIPLINES, Clinical trials < THERAPEUTICS

SCHOLARONE™
Manuscripts

Personalized immunotherapy in sepsis: a scoping review protocol

M.A. Slim[1,2]*, N. van Mourik[1,2]*, J.C. Dionne[3,4,5,6], S.J.W. Oczkowski[3,4,5], M.G. Netea[7], P. Pickkers[8], E. J. Giamarellos-Bourboulis[9], M.C.A. Müller[2], T. van der Poll[1,10], W.J. Wiersinga[1,10], A.P.J. Vlaar[2], L.A. van Vught[1,2]

* Both authors contributed equally.

Affiliations
[1] Center for Experimental and Molecular Medicine, Amsterdam University Medical, University of Amsterdam, Amsterdam, The Netherlands
[2] Department of Intensive Care, Amsterdam University Medical Centers, University of Amsterdam, Amsterdam, The Netherlands
[3] Department of Medicine, McMaster University, Hamilton, Canada.
[4] The Guidelines in Intensive Care Development and Evaluation (GUIDE) Group, Research Institute St. Joseph's Healthcare Hamilton, Hamilton, Canada.
[5] Department of Health Research Methods, Evidence, and Impact, McMaster University, Hamilton, Canada.
[6] Division of Gastroenterology, McMaster University, Hamilton, ON, Canada.
[7] Department of Internal Medicine and Radboud Center for Infectious Diseases, Radboud University Medical Center, Nijmegen, The Netherlands
[8] Department of Intensive Care Medicine, Radboud University Medical Center
[9] 4th Department of Internal Medicine, National and Kapodistrian University of Athens, Medical School, Athens, Greece
[10] Department of Internal Medicine, Division of Infectious Diseases, Amsterdam University Medical Centers, University of Amsterdam, Amsterdam, The Netherlands

Corresponding author
Marleen Slim; Department of Intensive Care Medicine, Amsterdam University Medical Center, Meibergdreef 9, Room G3-220, 1105 AZ Amsterdam, the Netherlands. E: m.a.slim@amsterdamumc.nl

Manuscript details

Journal:	BMJ Open
Article type:	Protocol
Word count abstract:	288 (out of 300)
Word count article:	1748 (out of 4000)
Table count:	6 (all in the supplement)
Reference count:	22

For peer review only - http://bmjopen.bmj.com/site/about/guidelines.xhtml

Abstract

Introduction. Sepsis, a life-threatening organ dysfunction syndrome occurring in the context of severe infections, remains a major burden on global health with high morbidity and high mortality rates. Despite recent advances in the understanding of its pathophysiology, the treatment of sepsis remains supportive of nature with few interventions specifically designed for treating this complex syndrome. The focus of sepsis trials has increasingly shifted towards targeting excessive inflammation and immunosuppression using immunomodulatory agents. However, it remains uncertain how to identify patients that could benefit from such treatment, whether treatments can be tailored to an individual's immune profile, or at which stage of the disease the intervention should be initiated. In this scoping review, we provide a comprehensive overview of current available literature on immunostimulatory and immunosuppressive therapies against sepsis.

Methods and analysis. The aim of this scoping review is to describe and summarize current literature evaluating immunotherapy in adult patients with sepsis. The review will be performed using the framework formulated by Arksey and O'Malley. A comprehensive literature and study collection will be executed by searching PubMed, Embase, Cochrane CENTRAL and ClinicalTrials.gov to identify clinical trials and cohort studies concerning immunotherapy in adult patients with sepsis. Screening will be performed independently and in duplicate by two reviewers who will also independently extract data into pre-specified spreadsheets. We will summarize evidence in tabular format with descriptive statistics. The reported evidence will convey knowledge on the types of immunotherapies studied, and currently being studied, in adult patients with sepsis.

Ethics and dissemination. Approval from a medical ethics committee is not required. Once completed, the review will be submitted for publication in a peer-reviewed journal. These results will be of value to clinicians and researchers with an interest in advancing sepsis care.

For peer review only - http://bmjopen.bmj.com/site/about/guidelines.xhtml

Article Summary

Strengths and limitations of this study

- This will be the first review using the scoping review methodology to systematically summarize which immunotherapies have been, and are currently being, studied in sepsis, this will highlight the gaps in in the field of immunotherapy in sepsis.
- In this review we will not only focus on immunotherapy alone, but also address studies that use a personalized approach to immunotherapy.
- In this review we will adhere to Preferred Reporting Items for Systematic Reviews and Meta-Analyses-Scoping Review (PRISMA) guidelines, conducting abstract screening, full-text screening and data extraction in independent duplication to facilitate greater inter-reviewer reliability.
- A potential limitation of this review lies within the heterogeneity between trials; sepsis is a heterogeneous syndrome and trials use trial-specific in-and exclusion criteria, leading to possible heterogeneity within the population studied.

For peer review only - http://bmjopen.bmj.com/site/about/guidelines.xhtml

Introduction

Rationale

Sepsis is defined as a life-threatening organ dysfunction syndrome caused by a dysregulated host response to an infection[1 2]. The global disease burden of sepsis remains high, with 48.9 million cases annually and almost 20% of all global deaths are sepsis-related[3]. Over the past few decades, significant progress in the understanding of the pathophysiology of sepsis has been made. Early diagnosis, timely antibiotic treatment and organ support are pivotal and are associated with a decrease in mortality rates[2]. Despite numerous efforts to date, there are few therapies which target the immunological basis of this life-threatening condition[4]. So far, treatment of sepsis mainly revolves around supportive care; administering antibiotics, fluids and vasopressors or initiating organ support.

During sepsis, the host response may be altered in multiple ways, explaining the highly heterogeneous clinical presentation, treatment responses and prognosis[5]. While the main pathophysiological cause of sepsis is often thought to be an excessive inflammatory immune response to an infection, it is now understood that a secondary immunosuppressive response often occurs concurrently[4-6]. Modulating the immune response to infection represents a promising treatment target in sepsis. However, over 100 clinical trials investigating the use of immunomodulatory agents in sepsis have been unsuccessful in demonstrating a benefit of immunomodulatory therapy[4 7]. One reason these trials may not have demonstrated a benefit is the use of a "one-size-fits-all" approach to immunotherapy. Sepsis is a clinically heterogeneous disease syndrome, with individual patients demonstrating varied clinical responses to infections. This suggests that personalized immunomodulatory treatment, tailored to an individual patient's immune profile, may be a better approach to treating patients suffering from sepsis[8]. Trials using personalized immunotherapy could increase the number of patients in the study with the potential to benefit from the administered treatment, and minimize the number of patients exposed to treatments which are unlikely to help or even cause harm[8]. The first step towards implementation of personalized medicine using immunomodulatory treatment is to provide a

For peer review only - http://bmjopen.bmj.com/site/about/guidelines.xhtml

structured and in-depth overview of currently available evidence on immunotherapy in the treatment of sepsis.

Study aim

The purpose of this scoping review is to describe and summarize current literature evaluating immunotherapy in adult patients with sepsis and the extent to which these trials used a personalized medicine approach.

Methods and analysis

The present study will be a scoping review performed using previously defined methods[9 10]. The model by Arksey and O'Malley defines a five-stage methodological framework, which includes; (1) identifying the research question, (2) identifying relevant studies, (3) study selection, (4) charting the data, and (5) collating, summarizing and reporting the results[9].

Stage 1: Identifying the research question

The following research questions will be addressed:

Primary objectives

1. Which immunotherapies have been, or are currently being, studied in adult patients with sepsis?
2. What are the study characteristics and patient populations of trials on immunotherapy in adult patients with sepsis?
3. How have clinical trials and cohort studies on sepsis used a personalized immunotherapy approach (including clinical phenotypes; immunoprofiling: cell populations, protein biomarkers and/or immune assays), and have these approaches changed over time?

Stage 2: Identifying relevant studies

For peer review only - http://bmjopen.bmj.com/site/about/guidelines.xhtml

A comprehensive literature search will be conducted using PubMed, Embase, Cochrane CENTRAL and ClinicalTrials.gov. Inclusion criteria are 1) clinical trials or cohort studies (including case control studies and observational cohorts) 2) investigating immunomodulatory therapies in 3) adult (≥ 16 years) patients with sepsis. All studies addressing therapies with an established immunomodulatory effect will be included. Studies addressing therapies with a hypothesized immunomodulatory effect (e.g. antibiotics, fluids, statins, immunonutrition, β-blockers, vasopressors) will be included, but only if the study investigates the immunomodulatory effects of the treatment. After completion of the initial literature search, we will use a snowball approach to find additional studies of interest[11]. All articles must contain full text written in English or Dutch. Exclusion criteria are 1) case studies, 2) animal studies and 3) studies in healthy volunteers. Systematic reviews and meta-analyses will also be excluded, but searched for potentially relevant references.

Search keywords include sepsis, immunotherapy, precision medicine, immunosuppression, anti-inflammatory agents, corticosteroid, anticoagulants, cytokines, granulocyte colony-stimulating factor, immunoglobulins, programmed cell death 1 receptor, Toll-like receptors, Polymyxin B, thymosin, protein C, mesenchymal stem cells, anti-endotoxin compound and extracorporeal blood removal. Articles retrieved from the database searches will be imported into Rayyan[12], an open-source program, to organize and facilitate screening of the search results. The full search strategies can be found in the **Supplementary Methods and Supplementary Table 1**. Prior to publication the search will be repeated to enable the inclusion of recently published studies.

Stage 3: Study selection

We will approach the study selection systematically using the inclusion and exclusion criteria described above, providing consistency in the decision-making regarding article selection. Screening will be performed independently and in duplicate by two reviewers (MS, NvM). The first step will be to screen all included articles in Rayyan on title and abstract. Disagreements at the trial/abstract stage will be

For peer review only - http://bmjopen.bmj.com/site/about/guidelines.xhtml

resolved by discussion between the two reviewers. When uncertainty or disagreement remains, a third reviewer (LvV) will be consulted to make a final decision regarding inclusion of the study. Final inclusion will be determined based on the analysis of the full text of any papers included by either reviewer during title/abstract screening stage. Disagreements at the full-text stage will be resolved in the same manner as described above at the title/abstract stage.

Stage 4: Charting the data

Data to be charted include:

1. Author(s), year of publication, study location;
2. Aim(s) of the study;
3. Study population, in- and exclusion criteria, number of patients;
4. Study design;
5. The type of immunotherapy and comparator (if any) used;
6. Main findings and outcome measures, including measured immune profiles;
7. If a personalized approach was used, and if so, which approach;
8. Limitations of the study stated by its authors;
9. Quality of evidence.

The quality of evidence will be critically appraised using the RoB 2 tool (a revised Cochrane risk of bias tool for randomized trials)[13] and the Newcastle Ottawa Quality Assessment Scale for Cohort Studies[14].

Two reviewers (MS, NvM) will independently extract data into pre-specified spreadsheets (**Supplementary Table 2 till 5**), separating data extracted from randomized controlled trials and observational controlled studies in order to better account for selection bias and confounding factors. At regular intervals, extracted data from the included articles in this framework will be compared in

For peer review only - http://bmjopen.bmj.com/site/about/guidelines.xhtml

order to ensure interrater reliability. Any disagreements or inconsistencies will be resolved by discussion, or, when uncertainty or disagreement remains, a third reviewer (LvV) will be consulted.

Stage 5: collating, summarizing and reporting the results

We will present an overview of the reviewed literature in tabular format with descriptive statistics. In addition, when possible, we will analyze treatment groups including (but not restricted to) strategies that modulate excessive inflammation (treatments targeting the innate immune response, complement, immunothrombosis and endothelial dysfunction, pleiotropic drugs with immunomodulatory effects, immunonutrition, supportive treatments with immunomodulatory effects, and non-pharmalogical immunomodulatory strategies) and strategies aiming at immune stimulation (immunomodulatory cytokines and growth factors, intravenous immunoglobulins, mesenchymal stem cells, and immune checkpoint inhibitors). Furthermore, we will present an overview of the trials currently recruiting or being studied in a supplementary table (**Supplementary Table 6**).

Patient and public involvement

No patient and/or public involved.

Ethics and dissemination

As this is scoping review, approval from a medical ethics committee is not required. Once completed, the review will be submitted for publication in a peer-reviewed journal.

Discussion

This scoping review will provide structured and detailed information on the types of immunotherapies studied in adult patients with sepsis. It will provide insight into how immunomodulatory trials have been conducted, and to what extent a personalized approach to immunotherapy has been used. An

For peer review only - http://bmjopen.bmj.com/site/about/guidelines.xhtml

overview of potential immunostimulatory and immunosuppressive treatments will be given, highlighting gaps in the field of sepsis treatment, and identifying strategies to study personalized immunotherapies in adult patients with sepsis.

In this review we will provide background on pathophysiological processes targeted within the investigated therapies. Compared to previous reviews on this topic[5 7 15-18], which mainly describe current advances in a narrative way, we here use a systematic approach to identify and summarize the evidence for immunomodulatory agents. In addition, the current review will add to the field of sepsis treatment by focusing on the evidence of personalized therapy.

This review will have several methodological strengths. First, as this will be a scoping review, the broad topic of immunotherapy in sepsis in which many different study designs might be applicable can be addressed in a complete and structured manner[9]. Second, we will systematically assess the risk of systematic errors via bias risk assessments, which will provide robustness of our results and conclusions. Third, we will focus on studies that used a personalized approach to immunotherapy, which is proposed to be the future of sepsis research[19 20].

A potential limitation of this review lies within the heterogeneity between trials. However, this is inherent when studying clinical trials with strict in- and exclusion criteria and is probably a good reflection of daily clinical practice in treating sepsis patients. Although inevitable, different criteria for sepsis have been used over time[2], leading to heterogeneity in the population included. Another limitation lies within the trials using a personalized approach to immunotherapy, since reliable biomarkers selecting the appropriate patient population that may most likely benefit from the treatment investigated, are still being studied[21 22]. However, systematically reviewing the studies investigating personalized immunotherapy will still add knowledge to the future sepsis management.

In conclusion, finding suitable treatment options for sepsis is crucial for improving the patient's outcome. Summarizing which immunotherapies have been, and are currently being, studied will highlight the gaps in in the field of immunotherapy in sepsis. This will be essential to improve further knowledge in the treatment of sepsis.

For peer review only - http://bmjopen.bmj.com/site/about/guidelines.xhtml

Author contributions

MS, TvdP, WJW, AV and LvV formed the study and outlined the proposal. This protocol, including the supplement, was written by MS, NM and LvV. MS, NvM, JD, SO, MGN, MM, AV and LvV have made a substantive intellectual contribution to the design of the protocol, the study aim, and research questions. They jointly developed the search strategy and data extraction framework. MS, NvM, JD, SO, MGN, PP, EGB, MM, TvdP, WJW, AV and LvV edited the manuscript and approved the final version.

Funding statement

MS, NvM, MGN, PP, EGB, TvdP, WJW and AV are supported by the European Commission (Horizon 2020, ImmunoSep, grant number 847422). WJW is supported by the Dutch Organization for Scientific Research (Nederlandse Organisatie voor Wetenschappelijk Onderzoek; VIDI grant 91716475). LvV is supported by the Netherlands Organization for Scientific Research (VENI grant 09150161910033). The funding sources had no involvement in the study design, in the writing of the protocol and in the decision to submit the article for publication.

Competing interests statement

SO reports grants and/or contracts from Physician Services Incorporated Ontario and Canadian Institutes of Health Research, and support from the American Thoracic Society and the European Respiratory Society. MGN reports an ERC Advanced Grant, a Spinoza Grant and is on the scientific advisory board of SOBI. PP is on the DSMB of the IL-7 trial 'ILEAD'. EGB reports grants and/or contracts from Abbott CH, bioMerieux, MSD, Sobi, and ThermoFisher BRAHMS. MM is on the DSMB of the PACER trial and the National Dutch Guideline on anticoagulation in COVID-19. TvdP reports project grants from the European Commission and is on the DSMBs of RECAP-CAP, SuDDICU and the CONFIDENT trial. WJW reports a project grant from ZonMW/NWO and receives consulting fees from Merck, Sobi and GlaxoSmithKline. AV receives consulting fees from InflaRx. The other authors have nothing to declare.

For peer review only

For peer review only - http://bmjopen.bmj.com/site/about/guidelines.xhtml

References

1. Seymour CW, Liu VX, Iwashyna TJ, et al. Assessment of Clinical Criteria for Sepsis: For the Third International Consensus Definitions for Sepsis and Septic Shock (Sepsis-3). *JAMA* 2016;315(8):762-74. doi: 10.1001/jama.2016.0288 [published Online First: 2016/02/24]
2. Singer M, Deutschman CS, Seymour CW, et al. The Third International Consensus Definitions for Sepsis and Septic Shock (Sepsis-3). *JAMA* 2016;315(8):801-10. doi: 10.1001/jama.2016.0287 [published Online First: 2016/02/24]
3. Rudd KE, Johnson SC, Agesa KM, et al. Global, regional, and national sepsis incidence and mortality, 1990–2017: analysis for the Global Burden of Disease Study. *The Lancet* 2020;395(10219):200-11. doi: 10.1016/s0140-6736(19)32989-7
4. van der Poll T, van de Veerdonk FL, Scicluna BP, et al. The immunopathology of sepsis and potential therapeutic targets. *Nat Rev Immunol* 2017;17(7):407-20. doi: 10.1038/nri.2017.36 [published Online First: 2017/04/25]
5. Steinhagen F, Schmidt SV, Schewe JC, et al. Immunotherapy in sepsis - brake or accelerate? *Pharmacol Ther* 2020;208:107476. doi: 10.1016/j.pharmthera.2020.107476 [published Online First: 2020/01/14]
6. Rubio I, Osuchowski MF, Shankar-Hari M, et al. Current gaps in sepsis immunology: new opportunities for translational research. *The Lancet Infectious Diseases* 2019;19(12):e422-e36. doi: 10.1016/s1473-3099(19)30567-5
7. Davies R, O'Dea K, Gordon A. Immune therapy in sepsis: Are we ready to try again? *J Intensive Care Soc* 2018;19(4):326-44. doi: 10.1177/1751143718765407 [published Online First: 2018/12/06]
8. Marshall JC. Why have clinical trials in sepsis failed? *Trends Mol Med* 2014;20(4):195-203. doi: 10.1016/j.molmed.2014.01.007 [published Online First: 2014/03/04]
9. Arksey H, O'Malley L. Scoping studies: towards a methodological framework. *International Journal of Social Research Methodology* 2005;8(1):19-32. doi: 10.1080/1364557032000119616
10. JBI Scoping Review Network - Resources [Available from: https://jbi.global/scoping-review-network/resources accessed Accessed October 18, 2021.
11. Wohlin C. Guidelines for snowballing in systematic literature studies and a replication in software engineering. Proceedings of the 18th International Conference on Evaluation and Assessment in Software Engineering - EASE '14, 2014:1-10.
12. Ouzzani M, Hammady H, Fedorowicz Z, et al. Rayyan-a web and mobile app for systematic reviews. *Syst Rev* 2016;5(1):210. doi: 10.1186/s13643-016-0384-4 [published Online First: 2016/12/07]
13. Risk of bias tool - RoB 2 tool [Available from: https://sites.google.com/site/riskofbiastool/welcome/rob-2-0-tool?authuser=0 accessed Accessed October 18, 2021.
14. Newcastle - Ottawa Quality Assessment Scale - Cohort Studies [Available from: https://www.ncbi.nlm.nih.gov/books/NBK115843/bin/appe-fm3.pdf accessed Accessed on October 18, 2021.
15. Hotchkiss RS, Monneret G, Payen D. Sepsis-induced immunosuppression: from cellular dysfunctions to immunotherapy. *Nat Rev Immunol* 2013;13(12):862-74. doi: 10.1038/nri3552 [published Online First: 2013/11/16]
16. Hotchkiss RS, Monneret G, Payen D. Immunosuppression in sepsis: a novel understanding of the disorder and a new therapeutic approach. *The Lancet Infectious Diseases* 2013;13(3):260-68. doi: 10.1016/s1473-3099(13)70001-x
17. Patil NK, Bohannon JK, Sherwood ER. Immunotherapy: A promising approach to reverse sepsis-induced immunosuppression. *Pharmacol Res* 2016;111:688-702. doi: 10.1016/j.phrs.2016.07.019 [published Online First: 2016/07/30]

For peer review only - http://bmjopen.bmj.com/site/about/guidelines.xhtml

18. van der Poll T. Immunotherapy of sepsis. *The Lancet Infectious Diseases* 2001;1(3):165-74. doi: 10.1016/s1473-3099(01)00093-7

19. Rello J, Valenzuela-Sanchez F, Ruiz-Rodriguez M, et al. Sepsis: A Review of Advances in Management. *Adv Ther* 2017;34(11):2393-411. doi: 10.1007/s12325-017-0622-8 [published Online First: 2017/10/13]

20. Rello J, van Engelen TSR, Alp E, et al. Towards precision medicine in sepsis: a position paper from the European Society of Clinical Microbiology and Infectious Diseases. *Clin Microbiol Infect* 2018;24(12):1264-72. doi: 10.1016/j.cmi.2018.03.011 [published Online First: 2018/03/28]

21. van Engelen TSR, Wiersinga WJ, Scicluna BP, et al. Biomarkers in Sepsis. *Crit Care Clin* 2018;34(1):139-52. doi: 10.1016/j.ccc.2017.08.010 [published Online First: 2017/11/19]

22. Peters van Ton AM, Kox M, Abdo WF, et al. Precision Immunotherapy for Sepsis. *Front Immunol* 2018;9:1926. doi: 10.3389/fimmu.2018.01926 [published Online First: 2018/09/21]

For peer review only

For peer review only - http://bmjopen.bmj.com/site/about/guidelines.xhtml

Supplementary online content

Personalized immunotherapy in sepsis: a scoping review protocol

M.A. Slim*, N. van Mourik*, J.C. Dionne, S.J.W. Oczkowski, M.G. Netea, P. Pickkers, E. J. Giamarellos-Bourboulis, M.C.A. Müller, T. van der Poll, W.J. Wiersinga, A.P.J. Vlaar, L.A. van Vught
* Both authors contributed equally.

For peer review only

Content	Page
Supplementary Methods	2
Supplementary Table 1.	4
Supplementary Table 2.	5
Supplementary Table 3.	6
Supplementary Table 4.	7
Supplementary Table 5.	8
Supplementary Table 6.	9

For peer review only - http://bmjopen.bmj.com/site/about/guidelines.xhtml

Supplementary Methods. The full search string

PubMed
2021 May 04

2444 hits:
("Sepsis"[Majr] OR sepsis[tiab] OR septic[tiab])

AND

("Immunotherapy"[Mesh] OR "Precision Medicine"[Mesh] OR "Immunosuppression"[Mesh] OR "Anti-Inflammatory Agents/therapeutic use"[Mesh] OR "Anti-Infective Agents/therapeutic use"[Mesh] OR "Glucocorticoids"[Mesh] OR "Hydroxycorticosteroids"[Mesh] OR "Anticoagulants/therapeutic use"[Mesh] OR "Cytokines/antagonists and inhibitors"[Mesh] OR "Cytokines/therapeutic use"[Mesh] OR "Granulocyte Colony-Stimulating Factor/therapeutic use"[Mesh] OR "Granulocyte-Macrophage Colony-Stimulating Factor/therapeutic use"[Mesh] OR "Immunoglobulins, Intravenous/therapeutic use"[Mesh] OR "Programmed Cell Death 1 Receptor/therapeutic use"[Mesh] OR "Toll-Like Receptors/therapeutic use"[Mesh] OR "Polymyxin B/therapeutic use"[Mesh] OR "Thymosin/therapeutic use"[Mesh] OR "Protein C/therapeutic use"[Mesh] OR "Mesenchymal Stem Cells"[Mesh] OR immunotherap*[tiab] OR interleukin*[tiab] OR interferon[tiab] OR toll-like-receptor agonist*[tiab] OR anti-endotoxin compound*[tiab] OR blocking complement*[tiab] OR activated vitamin C[tiab] OR mesenchymal stem cell*[tiab] OR immune checkpoint inhibitor*[tiab] OR extracorporeal blood removal[tiab] OR corticosteroid*[tiab])

AND

("Clinical Trial" [Publication Type] OR clinical trial*[tiab] OR randomized trial*[tiab] OR randomized trial*[tiab] OR controlled trial*[tiab])

NOT

(("Child"[Mesh] OR "Infant"[Mesh] OR "Pediatrics"[Mesh] OR child*[tiab] OR infan*[tiab] OR newborn*[tiab] OR neonat*[tiab] OR baby[tiab] OR babies[tiab] OR pediatr*[tiab] OR paediatr*[tiab]) NOT "Adult"[Mesh])

NOT

("Editorial"[Publication Type] OR "Letter"[Publication Type] OR "News"[Publication Type] OR "Comment"[Publication Type] OR "Review" [Publication Type] OR "Case Reports"[Publication Type] OR "letter*"[Title] OR "comment*"[Title] OR editorial[ti])

NOT

(("Animals"[MeSH Terms] OR "Animal Experimentation"[MeSH Terms] OR "animals, laboratory"[MeSH Terms] OR "models, animal"[MeSH Terms] OR "animal*"[Title/Abstract] OR "rat"[Title/Abstract] OR

For peer review only

"rats"[Title/Abstract] OR "mice"[Title/Abstract] OR "mouse"[Title/Abstract] OR "dog"[Title/Abstract] OR "dogs"[Title/Abstract] OR "pig"[Title/Abstract] OR "pigs"[Title/Abstract] OR "cow"[Title/Abstract] OR "cows"[Title/Abstract] OR "monkey"[Title/Abstract] OR "monkeys"[Title/Abstract] OR "horse"[Title/Abstract] OR "horses"[Title/Abstract] OR "sheep"[Title/Abstract] OR "ovine"[Title/Abstract] OR "lamb"[Title/Abstract] OR "lambs"[Title/Abstract] OR "goat*"[Title/Abstract] OR "swine"[Title/Abstract] OR "porcine"[Title/Abstract] OR "pup"[Title/Abstract] OR "pups"[Title/Abstract] OR "canine"[Title/Abstract] OR "bitch*"[Title/Abstract] OR "beagle"[Title/Abstract] OR "feline"[Title/Abstract] OR "rodent*"[Title/Abstract] OR "rabbit*"[Title/Abstract] OR "murine"[Title/Abstract] OR "ape"[Title/Abstract] OR "apes"[Title/Abstract] OR "gorilla"[Title/Abstract] OR "gorillas"[Title/Abstract] OR "catfish"[Title/Abstract]) NOT "Humans"[MeSH Terms])

Cochrane Central Register of Controlled Trials
Issue 4 of 12, April 2021

ID Search Hits

#1 (sepsis or septic):ti,ab,kw 13698

#2 (immunotherap* OR interleukin* OR interferon OR toll-like-receptor agonist* OR anti-endotoxin compound* OR blocking complement* OR activated vitamin C OR mesenchymal stem cell* OR immune checkpoint inhibitor* OR extracorporeal blood removal OR corticosteroid*):ti,ab,kw 67451

#3 (precision medicine OR immunosuppress* OR anti-nflammatory agent* OR anticoagulant* OR cytokines OR granulocyte colon stimulating factor* OR granulocyte macrophage colony-stimulating factor OR immunoglobulin* OR programmed cell death 1 receptor* OR Toll-Like Receptors OR polymyxin B OR thymosin OR protein OR anti-infective agent* OR glucocorticoid* OR hydroxycorticosteroid* OR mesenchymal stem cell*):ti,ab,kw 126191

#4 #2 or #3 171251

#5 #1 and #4 in Trials 3246

ClinicaTrials.gov
2021 May 04
1309 Studies found for: Interventional Studies | sepsis | Adult, Older Adult

EMBASE (Ovid):
Database(s): Embase Classic+Embase 1947 to 2021 May 03

For peer review only

Supplementary Table 1. Search Strategy EMBASE:

#	Searches	Results
**1**	exp *sepsis/dt, th or (sepsis or septic).ti,ab,kw.	233069
**2**	exp immunotherapy/ or personalized medicine/ or exp immunosuppressive treatment/	497119
**3**	exp antiinflammatory agent/dt, th or exp antiinfective agent/dt, th or drotrecogin/ or exp corticosteroid/dt, th or exp anticoagulant agent/dt, th or exp cytokine/dt or granulocyte colony stimulating factor/dt or granulocyte macrophage colony stimulating factor/dt or exp immunoglobulin/dt, th or programmed death 1 receptor/dt or toll like receptor/dt or polymyxin B/ or thymosin/dt or exp mesenchymal stem cell/	1908414
**4**	((immunotherap* or interleukin* or interferon or toll-like-receptor agonist* or anti-endotoxin compound* or blocking complement* or activated vitamin C or mesenchymal stem cell* or immune checkpoint inhibitor* or extracorporeal blood removal or corticosteroid*) and (treat* or therap*)).ti,ab,kw.	532386
**5**	2 or 3 or 4	2556542
**6**	exp clinical trial/ or controlled clinical trial/ or controlled study/ or randomized controlled trial/	8934963
**7**	((randomized or randomised or controlled) adj3 trial*).ti,ab,kw.	623934
**8**	clinical trial*.ti,ab,kw.	600136
**9**	6 or 7 or 8	9356625
**10**	1 and 5 and 9	18560
**11**	(exp child/ or exp pediatrics/ or (child* or infan* or newborn* or neonat* or baby or babies or pediatr* or paediatr*).ti,ab.) not adult/	3086878
**12**	10 not 11	15898
**13**	(exp animal/ or exp animal experiment/ or exp animal model/ or exp veterinary medicine/ or (animal* or monkey* or sheep or ovine or lamb or lambs or goat* or pig or pigs or swine or porcine or pup or pups or dog or dogs or canine or bitch* or beagle or feline or rodent* or rabbit* or rat or rats or mice or mouse or murine or cow or cows or horse or horses or ape or apes or gorilla or gorillas).ti,ab,kw.) not human/	6558145
**14**	12 not 13	12416
**15**	letter/ or editorial/ or note/ or case report/ or conference paper/ or "review"/ or (letter or comment* or editorial or case report).ti.	8552442
**16**	14 not 15	9437

Supplementary Table 2. Data Abstraction Table – Randomized controlled trials – "Targeting (excessive) inflammation"

	Innate immune response	Complement	Immunomodulation / endothelial dysfunctions	Immunomodulation by pleiotropic drugs	Immunonutrition	Immunomodulation by supportive treatments	Non-pharmalogical immunomodulatory strategies
Author(s)							
Year							
Location							
Aim(s)							
Population							
Number of patients							
Design							
Intervention (dose, frequency, duration)							
Comparator (if any)							
Analyses (crude and adjusted)							
Main findings							
Measured immune profiles							
Personalized medicine; If so, how?							
Limitations as stated by author							
Quality of evidence							

Supplementary Table 3. Data Abstraction Table – Randomized controlled trials – "Immune stimulation"

	Immunostimulatory cytokines and growth factors	Intravenous immunoglobulins	Mesenchymal stem cells	Immune checkpoint inhibitors
**Author(s)**				
**Year**				
**Location**				
**Aim(s)**				
**Population**				
**Number of patients**				
**Design**				
**Intervention (dose, frequency, duration)**				
**Comparator (if any)**				
**Analyses (crude and adjusted)**				
**Main findings**				
**Measured immune profiles**				
**Personalized medicine; If so, how?**				
**Limitations as stated by author**				
**Quality of evidence**				

Supplementary Table 4. Data Abstraction Table – Observational controlled studies – "Targeting (excessive) inflammation"

	Innate immune response	Complement	Immunomodulation / endothelial dysfunctions	Immunomodulation by pleiotropic drugs	Immunonutrition	Immunomodulation by supportive treatments	Non-pharmalogical immunomodulatory strategies
Author(s)							
Year							
Location							
Aim(s)							
Population							
Number of patients							
Design							
Intervention (dose, frequency, duration)							
Comparator (if any)							
Analyses (crude and adjusted)							
Main findings							
Measured immune profiles							
Personalized medicine; If so, how?							
Limitations as stated by author							
Quality of evidence							

For peer review only - http://bmjopen.bmj.com/site/about/guidelines.xhtml

Supplementary Table 5. Data Abstraction Table – Observational controlled studies – "Immune stimulation"

	Immunostimulatory cytokines and growth factors	Intravenous immunoglobulins	Mesenchymal stem cells	Immune checkpoint inhibitors
Author(s)				
Year				
Location				
Aim(s)				
Population				
Number of patients				
Design				
Intervention (dose, frequency, duration)				
Comparator (if any)				
Analyses (crude and adjusted)				
Main findings				
Measured immune profiles				
Personalized medicine; If so, how?				
Limitations as stated by author				
Quality of evidence				

For peer review only - http://bmjopen.bmj.com/site/about/guidelines.xhtml

Supplementary Table 6. Data Abstraction Table – trials currently recruiting or being studied

	Trial
**Investigator(s)**	
**Start Date**	
**Location**	
**Aim(s)**	
**Outcome(s)**	
**Population**	
**Number of patients**	
**Design**	
**Intervention (dose, frequency, duration)**	
**Comparator (if any)**	
**Measured immune profiles**	
**Personalized medicine; If so, how?**	

er review only

PRISMA-P (Preferred Reporting Items for Systematic review and Meta-Analysis Protocols) 2015 checklist: recommended items to address in a systematic review protocol*

Section and topic	Item No	Checklist item	Information reported YES	NO		Page numbers
**ADMINISTRATIVE INFORMATION**			**YES**	**NO**		**Page numbers**
Title:						
Identification	1a	Identify the report as a protocol of a systematic review	☒	☐		See page 1.
Update	1b	If the protocol is for an update of a previous systematic review, identify as such	☐	☒	N/A	
Registration	2	If registered, provide the name of the registry (such as PROSPERO) and registration number	☐	☒	This review could not be registered in the international prospective register of systematic reviews (PROSPERO) because they do not accept scoping reviews.	
Authors:						
Contact	3a	Provide name, institutional affiliation, e-mail address of all protocol authors; provide physical mailing address of corresponding author	☒	☐		See page 1.
Contributions	3b	Describe contributions of protocol authors and identify the guarantor of the review	☒	☐		See page 10.
Amendments	4	If the protocol represents an amendment of a previously completed or published protocol, identify as such and list changes; otherwise, state plan for documenting important protocol amendments	☐	☒	N/A	
Support:						
Sources	5a	Indicate sources of financial or other support for the review	☐	☒	There was no financial support for this review. For the financial support of the researchers see page 10.	
Sponsor	5b	Provide name for the review funder and/or sponsor	☐	☒	N/A	
Role of sponsor or funder	5c	Describe roles of funder(s), sponsor(s), and/or institution(s), if any, in developing the protocol	☐	☒	N/A	

For peer review only - http://bmjopen.bmj.com/site/about/guidelines.xhtml

**INTRODUCTION**						
Rationale	6	Describe the rationale for the review in the context of what is already known	☒	☐		See page 4.
Objectives	7	Provide an explicit statement of the question(s) the review will address with reference to participants, interventions, comparators, and outcomes (PICO)	☐	☒		
**METHODS**						
Eligibility criteria	8	Specify the study characteristics (such as PICO, study design, setting, time frame) and report characteristics (such as years considered, language, publication status) to be used as criteria for eligibility for the review	☒	☐		See page 6.
Information sources	9	Describe all intended information sources (such as electronic databases, contact with study authors, trial registers or other grey literature sources) with planned dates of coverage	☒	☐		See page 6, supplement pages 2-4.
Search strategy	10	Present draft of search strategy to be used for at least one electronic database, including planned limits, such that it could be repeated	☒	☐		See supplement pages 2-4.
Study records:						
Data management	11a	Describe the mechanism(s) that will be used to manage records and data throughout the review	☒	☐		See page 6.
Selection process	11b	State the process that will be used for selecting studies (such as two independent reviewers) through each phase of the review (that is, screening, eligibility and inclusion in meta-analysis)	☒	☐		See pages 6-7.
Data collection process	11c	Describe planned method of extracting data from reports (such as piloting forms, done independently, in duplicate), any processes for obtaining and confirming data from investigators	☒	☐		See pages 7-8.
Data items	12	List and define all variables for which data will be sought (such as PICO items, funding sources), any pre-planned data assumptions and simplifications	☒	☐		See supplement pages 5-9.
Outcomes and prioritization	13	List and define all outcomes for which data will be sought, including prioritization of main and additional outcomes, with rationale	☐	☒	Since this is a scoping review, all study specific outcomes will evaluated.	
Risk of bias in individual studies	14	Describe anticipated methods for assessing risk of bias of individual studies, including whether this will be done at the outcome or study level, or both; state how this information will be used in data synthesis	☒	☐		See page 7.
Data synthesis	15a	Describe criteria under which study data will be quantitatively synthesised	☐	☒		

For peer review only

	15b	If data are appropriate for quantitative synthesis, describe planned summary measures, methods of handling data and methods of combining data from studies, including any planned exploration of consistency (such as I^2, Kendall's τ)	☐	☒	
	15c	Describe any proposed additional analyses (such as sensitivity or subgroup analyses, meta-regression)	☐	☒	
	15d	If quantitative synthesis is not appropriate, describe the type of summary planned	☒	☐	See page 8.
Meta-bias(es)	16	Specify any planned assessment of meta-bias(es) (such as publication bias across studies, selective reporting within studies)	☐	☒	
Confidence in cumulative evidence	17	Describe how the strength of the body of evidence will be assessed (such as GRADE)	☐	☒	

*** It is strongly recommended that this checklist be read in conjunction with the PRISMA-P Explanation and Elaboration (cite when available) for important clarification on the items. Amendments to a review protocol should be tracked and dated. The copyright for PRISMA-P (including checklist) is held by the PRISMA-P Group and is distributed under a Creative Commons Attribution Licence 4.0.**

From: Shamseer L, Moher D, Clarke M, Ghersi D, Liberati A, Petticrew M, Shekelle P, Stewart L, PRISMA-P Group. Preferred reporting items for systematic review and meta-analysis protocols (PRISMA-P) 2015: elaboration and explanation. BMJ. 2015 Jan 2;349(jan02 1):g7647.

For peer review only
